# Viewing social isolation as a complex dynamical system: A theoretical and computational framework

**DOI:** 10.1038/s44277-025-00051-y

**Published:** 2025-12-09

**Authors:** Samuel J. Abplanalp, Joseph S. Maimone, Michael F. Green

**Affiliations:** 1 Desert Pacific Mental Illness Research, Education and Clinical Center, Veterans Affairs Greater Los Angeles Healthcare System, Los Angeles, CA USA; 2 Department of Psychiatry and Biobehavioral Sciences, David Geffen School of Medicine, University of California, Los Angeles, CA USA; 3 Department of Psychological and Brain Sciences, Boston University, Boston, MA USA; 4 Sargent College of Rehabilitation Sciences, Boston University, Boston, MA USA

**Keywords:** Signs and symptoms, Psychology

## Abstract

Social isolation is a major public health concern linked to increased risk for both psychiatric and physical health conditions. Yet despite the potential consequences of social isolation, our understanding of its nature and how it emerges and evolves over time remains limited. We propose that social isolation should be understood and analyzed as a complex dynamical system. First, we introduce core principles of dynamical systems theory and describe how they can be applied to better understand social isolation. Second, we formalize a dynamical systems model using differential equations. Third, we present simulations based on the differential equations showing how changes in system dynamics may increase or decrease the likelihood of individuals entering a state of social isolation. Fourth, we provide a brief simulation-recovery analysis demonstrating model parameter identifiability from intensive longitudinal data designs. Finally, we offer a simulated example of how intensive longitudinal data could be used to identify signs of transitions between healthy and isolated states. Overall, this framework, both theoretical and computational, helps elucidate the dynamic nature of social isolation and may ultimately inform empirical research and personalized interventions capable of identifying those at risk for transitioning into a state of isolation.

## Lay Summary

Social isolation, or having objectively few social relationships, is associated with psychiatric and physical health problems. However, it is unclear how social isolation develops in an individual. Here, we propose a theoretical and computational framework that aims to study how social isolation develops and changes over time. Ultimately, this framework may be able to help identify who is socially isolated and how, when, and why individuals transition into a chronic state of isolation.

## Introduction

Human beings are a social species. Interactions with friends, family, and acquaintances contribute positively to well-being and quality of life [1–3]. In fact, the pursuit and maintenance of social connection may serve an evolutionary function and appear “hardwired” into the brain [4, 5]. Lacking social connection may manifest in a variety of ways, but it is usually measured by social isolation and loneliness. Although social isolation and loneliness are related, they are not one in the same. Social isolation is defined as having objectively few social relationships, social roles, group memberships, and infrequent social interaction [6, 7]. Meanwhile, loneliness is a distressing subjective experience that results from perceived isolation or unmet need between preferred and actual social experience [7, 8]. Critically, individuals can be socially isolated and not experience loneliness, as well as experience loneliness and not be socially isolated.

A recent Surgeon General’s Advisory cited social isolation and loneliness as urgent public health issues [9]. Indeed, social isolation and loneliness are associated with a broad range of physical (e.g., Type 2 diabetes [10], pulmonary dysfunction [11]) and psychological (e.g., depression [12], suicidal behavior [13], psychosis [14]) health conditions and cost more than $160 billion annually [15, 16]. However, evidence is beginning to suggest that social isolation may have a more deleterious impact on overall health and mortality. For example, a study conducted using data from the UK Biobank showed that socially isolated individuals have a 26% increased likelihood of developing dementia after controlling for other key risk factors (e.g., cardiovascular disease, depression, genetics) [17]. In contrast, the association between dementia and loneliness was only present when depression was not accounted for in analyses [17]. Similarly, longitudinal studies indicate that the association between social isolation and risk of mortality is present even after accounting for sociodemographic and health-related variables [18, 19]; however, the association between loneliness and mortality risk may be largely attributed to the effects of other variables [18, 19]. Thus, social isolation—to a stronger degree than loneliness— appears to be an independent risk factor for numerous health conditions and early mortality.

Numerous variables are thought to underlie social isolation [20, 21]. Current conceptual models often identify variables at different levels of analysis that contribute to isolation, including variables at the psychological, behavioral, and societal levels [22, 23]. While these models are useful for illustrating the wide range of variables that can influence social isolation, they are incomplete as they do not inform us on the *dynamic* nature of social isolation. A substantial limitation of most existing models is that they conceptualize social isolation as a static end point rather than a process that unfolds over time. With that said, researchers have begun developing theoretical models of social isolation that aim to understand it as a dynamic process.

One study interviewed older adults in Singapore who live alone or have limited social interactions about what factors they believed lead to social isolation over time [24]. From these interviews, the authors derived a qualitative model of social isolation that aligns well with existing conceptual models [i.e., 22, 23] but adds a network of complex interrelated mechanisms that aims to elucidate the causal dynamics of social isolation [24]. In a separate study, data from the National Health and Aging Trends Study (NHATS) were used to track social isolation among a nationally representative sample of over 7,000 U.S.-based older adults every year for 10 years. Results showed that over a quarter of the sample evidenced year-to-year transitions between social integration and social isolation [25]. To our knowledge, this is the most intensive data available to demonstrate the dynamic nature of social isolation. Taken together, these two studies, although restricted to older adults, signal increasing emphasis on theoretical models of social isolation that center on better understanding its temporal dynamics and identifying modifiable risk factors. Being able to more precisely model the temporal dynamics of isolation and how it emerges and evolves over time can help reveal critical windows for early detection and more effective intervention.

To study how social isolation emerges and evolves over time, we can represent it as a *complex dynamical system*—a mathematical model that describes how variables evolve according to a determined “set of rules”. These rules can be formalized and subsequently expressed as differential equations, which provide a powerful tool for modeling social isolation because they can capture the precise representation of complex interactions and feedback mechanisms that drive social behavior. A critical advantage of mathematical models such as these compared to traditional hypothesis testing is their ability to incorporate and test incremental improvements over time. Mathematical models allow future research to create a more rigorous and testable framework, enabling more precise insights and more systematic examination of phenomena [26, 27].

We propose a theoretical and computational framework grounded in dynamical systems theory to understand how social isolation develops and persists over time. We conceptualize social isolation as one possible state— called *an attractor state* —within a dynamical system. In such a system, socially relevant processes at different levels of analysis such as social motivation, cognitive interpretations of social experiences, and external conditions (e.g., neighborhood factors) interact over time to shift the system into a specific state. For example, one state can be characterized by engagement and connection and another by withdrawal and isolation. By shifting the focus from static and linear measurement to a dynamical system approach, researchers can examine *who* is socially isolated and *how*, *when*, and *why* individuals transition into a chronic state of isolation.

This paper is organized as follows. First, we introduce social isolation as a complex dynamical system and identify processes that could serve as possible parameters. Second, we formalize this model using differential equations. Third, we present simulations showing how variations in parameter values and perturbations (i.e., disruptions to the state of the system) can produce qualitative shifts in system dynamics that make it more or less likely to transition into a different state. Fourth, we conduct a simulation-recovery analysis demonstrating model parameter identifiability. Fifth, we present a simulated, illustrative example of how researchers could use a dynamical systems perspective to understand and analyze early signs of state transitions. Finally, we discuss challenges that come with operationalizing social isolation as a complex dynamical system and how future research can address such challenges.

### Understanding social isolation as a complex dynamical system

**Fig. 1:**
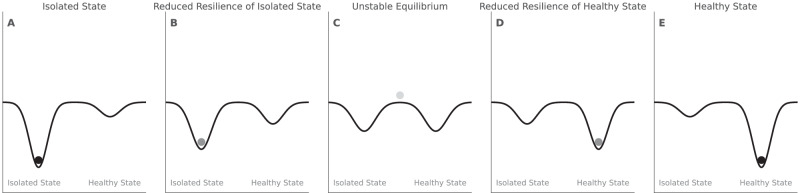
Changing Resiliency of a Dynamical System of Social Isolation. The resiliency of a hypothetical dynamical system of social isolation is linked to the strength of the attractor states, which is represented by landscapes and basins. The deeper the basin, the more resilient the attractor state. **A** The socially isolated state is highly resilient with a deep basin—small perturbations will not disturb the state. **B** The isolated state is losing resilience, as its basin is shallower and the healthy basin has appeared. Small perturbations would now take longer for the system to recover from. **C** The system is at a tipping point characterized by an unstable equilibrium and relatively shallow basins. Small perturbations in either direction could cause the system to tip into a different attractor state. **D** The system has tipped into the healthy state but with low resilience. Small perturbations would take longer to recover from or cause the system to shift back to the tipping point. **E** The healthy state is now highly resilient with a deep basin—small perturbations will not disturb the state.

Dynamical systems approaches are increasingly used in psychological science to model phenomena such as mental disorders [28], suicide [29], and substance use [30]. Following Fatimah et al., who framed substance use relapse as a latent process drawn toward two states (abstinence vs relapse) [30], we model social isolation as a latent process with two attractors: a healthy state and a socially isolated state. These states act as *alternative attractors* that the system can “settle into” depending on initial conditions and external inputs [31, 32]. Whether a system settles into a particular state is primarily governed by the system’s *resilience*^1^. From a psychological perspective, resilience refers to an individual’s capacity to overcome adversity or stress. In contrast, a systems-based definition of resilience is the ability of a system to recover from perturbations and return to its prior equilibrium state [34, 35]. Between the two attractor states is an intermediate state, which serves as a threshold or *tipping point* [36]. The intermediate state is intrinsically unstable—even minor perturbations will push the system away from it and toward one of the attractors [37, 38]. In the context of social isolation, individuals near the intermediate state may be more vulnerable to abruptly “tip” into the healthy or isolated state, which can be depicted using the idea of landscapes. Figure 1 presents a visualization of different hypothetical landscapes corresponding to social isolation. Here, the deeper the basin, the more resilient the attractor state, regardless of whether it is a socially isolated or non-isolated (i.e., healthy) state. Importantly, “socially isolated” versus “not isolated” are not literal categories; the latent social state is continuous, and the landscape can support either two basins or a single broad basin depending on person and context. Apparent switches in states reflect graded movement on a continuum rather than an all-or-none change. Moreover, for conceptual simplicity we define social isolated or healthy states as the two theoretical extremes of this continuum, one end of which clearly contributes to the deleterious effects of social isolation whereas the other clearly does not.

Parameters called *control variables* help determine the resilience of the system and whether individuals may tip into different states [39]. There are two types of control variables: *external* and *internal*^2^. External and internal variables could broadly map onto variables at different levels of analysis shown to be associated with social isolation. For our purposes, external variables refer to relatively stable environmental or structural conditions that influence whether the system permits multiple possible outcomes [40]. Examples of external variables could include the size and density of a person’s social network, geographic or economic barriers to social interaction, or the availability of community support systems. Internal variables refer to psychological or behavioral processes that shape how individuals respond to their social environment over time [40]. Examples of internal variables could include social motivation (e.g., social anhedonia), cognitive biases (e.g., hostile attribution bias), mood, or patterns of social engagement. Whereas external variables set the broad conditions under which social isolation might emerge, internal variables determine how likely someone is to move toward or remain in a given state by amplifying or damping responses to disruptions in the system [40].

Take the example of someone with a sparse social network (an external control variable) who has low social motivation (an internal control variable). Having a sparse social network makes it possible for the system to have an isolated attractor state. Meanwhile, low social motivation may lead to internal feedback loops—such as when reduced social motivation leads to social withdrawal, which further reduces social motivation. When external variables strongly shape the social environment and internal variables shape responses to the environment, even relatively small life events, like missing a social gathering or having a disagreement with a friend, may push the individual close to a tipping point. Once near this threshold, minor additional perturbations, or the ongoing internal feedback dynamics themselves, can lead to an abrupt shift into an isolated state. Larger life events, such as a breakup, relocation, or a pandemic, can accelerate this process or trigger abrupt transitions directly. Once the system settles into the isolated state, its self-reinforcing nature can make it difficult to reverse, even if the original cause of the tipping is removed.

Resilience of the isolated state may decrease over time. In the scenario described above, external conditions could be improved by gaining a new group of interconnected friends. Internal feedback loops could be improved by reducing the signal of social motivational deficits. After external and/or internal conditions improve, small positive perturbations could move the individual back toward the transitional state, making it possible for them to switch into a healthy state. It is important to note that the resilient dynamics that contribute to individuals transitioning into an isolated state may involve different processes than the resilient dynamics that are required for them to transition out of an isolated state [41].

## Methods

### Formalizing social isolation as a complex dynamical system

We introduce a general mathematical model grounded in a dynamical systems perspective on social isolation. Rather than specifying particular control variables as model parameters, we present a simplified framework that captures the essential dynamics underlying transitions between healthy and isolated states, as well as the resilience of these states. Our goal is not to account for every facet of social isolation, but to illustrate how core components of a dynamical system over time can be formalized.

To formalize our framework, we model social isolation as a stochastic dynamical system characterized by a potential landscape—that is, a mathematical surface that reflects the relative resilience of different states (readers can refer back to Fig. 1 for an illustration of landscapes) [37, 42]. Here, an individual’s social state over time is represented by a continuous state variable, denoted ${Z}_{t}$, which reflects the current position along a continuum of social connectedness. The system evolves under the influence of two forces: a deterministic component governed by a *potential function* and a stochastic component that captures random fluctuations [43].

#### The potential function

The deterministic dynamics are derived from a potential function $V(Z)$, which sets the structure of the system’s potential landscape. We chose to represent the potential function with the following form:

1 $$V\left(Z\right)=\frac{1}{4}{Z}^{4}-\frac{1}{2}a{Z}^{2}-{bZ}$$

This equation^3^ defines the topography of the landscape and reflects how different combinations of internal and external influences can give rise to one or more attractor states. In this formulation, $Z$ is the latent state variable; $a$ is the internal control variables, such as social motivation or cognitive biases; and $b$ is the external variables, such as social network density or structural adversity. When plotted, this potential function can produce a single well or two wells separated by a ridge, corresponding to one or two states, respectively. The minima of this function correspond to attractor states and the local maximum between them corresponds to an unstable intermediate state or tipping point [44].

#### Gradient dynamics

The system drifts to evolve toward states that minimize the potential energy [39]. This movement is governed by the negative gradient flow of the potential function, which represents the direction of steepest descent on the potential landscape:

2 $$\frac{{dV}(Z)}{{dZ}}={Z}^{3}-{aZ}-b$$

Thus, the deterministic component of the dynamics is described by:

3 $$\frac{d{Z}_{t}}{{dt}}=-\frac{{dV}}{{dZ}}=-{Z}_{t}^{3}+a{Z}_{t}+b$$

This equation determines how the system evolves over time in the absence of noise. In this case, the deterministic component ensures that the system moves “downhill” toward the valleys (healthy and isolated states) of the potential landscape and away from peaks (unstable states or tipping points), which we chose to be consistent with how we expect our attractor states to behave. This “downhill” movement is a general property of gradient systems and reflects a standard approach in dynamical systems theory for modeling how systems drift toward stable states over time.

#### Stochastic dynamics

To account for unpredictable life events, stressors, or fluctuations in the social environment, we include a stochastic term based on a Wiener process ${W}_{t}$, which introduces continuous random noise [45, 46]. The full stochastic differential equation (SDE) becomes:

4 $$d{Z}_{t}=\left({-Z}_{t}^{3}+a{Z}_{t}+b\right){dt}+\sigma d{W}_{t}$$

Here, $\sigma$ controls the magnitude of the noise. This SDE defines how the social state ${Z}_{t}$ evolves over time as a function of both deterministic structure and stochastic influences. The potential function $V(Z)$ determines the shape of the system’s attractors, while the parameters *a*, *b*, and *σ* control its responsiveness to change and vulnerability to tipping points.

This mathematical model provides a foundation for exploring how different combinations of parameters shape system behavior. Next, we present simulations illustrating how changes in internal and external dynamics, along with perturbations, can produce qualitative shifts in the system’s trajectory.

### Model simulations

**Fig. 2:**
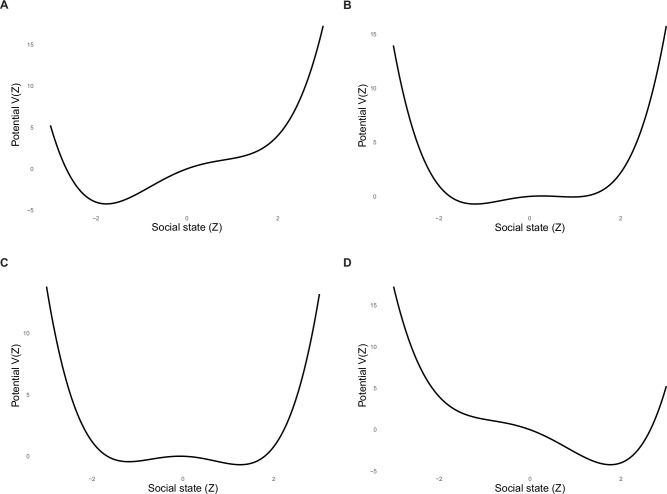
Simulations of Potential Landscapes. All simulations are based on the presented mathematical model. The values on the y-axes may be different based on the simulation because the potential landscapes represent qualitatively different states. **A** A potential landscape of a resilient socially isolated state. **B** A potential landscape of a destabilizing socially isolated state that is less resilient than A. **C** A potential landscape that is at a tipping point characterized by an unstable equilibrium. **D** A potential landscape of a resilient healthy state.

**Fig. 3:**
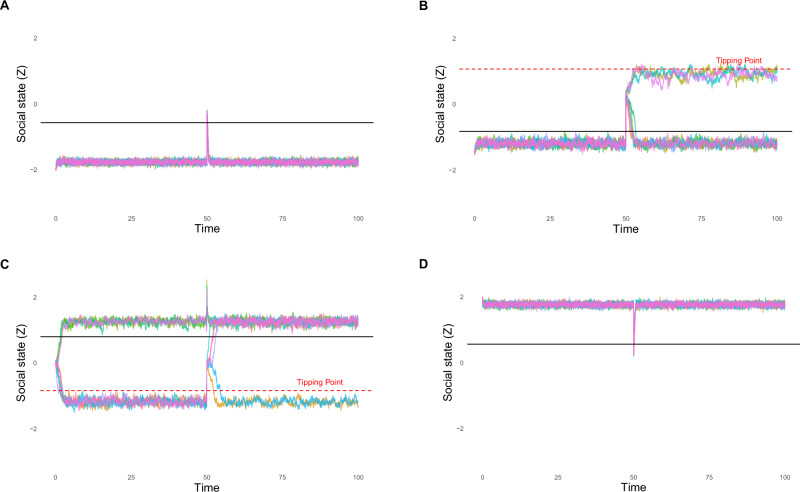
Simulations of Trajectories of the Potential Landscapes with and without Perturbation. The simulated trajectories correspond to 1 of the 4 simulated potential landscapes. Each plot was simulated with 10 trajectories in different colors to observe how the Wiener process (i.e., stochastic noise) can affect the system even under identical parameter values. The left side of the plot is without perturbation, and the right side of the plot (i.e., after a sharp increase or decrease in trajectories) is with perturbations. **A** Trajectory plot of the resilient socially isolated state. **B** Trajectory plot of the destabilizing socially isolated state. **C** Trajectory plot of the tipping state. **D** Trajectory plot of the resilient healthy state.

We present simulations of social isolation as a complex dynamical system using the mathematical model described above. These simulations depict how different combinations of internal and external control variables shape the system’s potential landscape and influence the resilience of the system. Figure 2 presents four example configurations of the system’s potential landscape corresponding to different regimes: (1) an isolated state, (2) a destabilizing state, (3) a tipping point state, and (4) a healthy state. Figure 3 shows trajectories of the system over time in each of these regimes, both with and without perturbations. We simulated perturbations as instantaneous exogenous shocks to the state variable ${Z}_{t}$ at a designated time point ${t}_{p}$ during numerical integration of the stochastic differential equation. Specifically, at $t={t}_{p}$, the system was perturbed by setting ${Z}_{{t}_{p}}={Z}_{{t}_{p}}+\Delta z$, enabling analysis of the system’s post-perturbation trajectory to assess whether it returned to the original attractor basin or crossed the tipping point into a different state. The tipping point appears as a dotted red line in the trajectories and is the unstable equilibrium of the fitted landscape, which is technically defined as the middle real root of $-{Z}^{3}+{aZ}+b=0$. For the tipping point to emerge, the social state must cross more than one basin; when only one real root exists (single-basin regime), the tipping point will not emerge.

Individual trajectories can be thought of as individual people, and differences in trajectories emerging from the stochastic process can be thought of as individual differences due to each person’s unique context. We used the Euler–Maruyama method in R (Version 4.4.1 [47]) to solve the stochastic differential equation [48].

#### Simulating an isolated state

We begin by simulating the system in a resilient isolated state^4^. The corresponding potential landscape (Fig. 2, Panel A) shows a deep attractor well on the left side of the plot, indicating strong resilience of the isolated state. The right well is either shallow or absent, making transitions toward greater connection unlikely.

The simulated trajectory without perturbation (Left side of Fig. 3, Panel A) confirms the state’s resiliency: the social state $Z$ remains near the isolated attractor over time, with minimal fluctuation. The solid black horizontal line marks the equilibrium point corresponding to the isolated state. When a large positive perturbation is introduced (Right side of Fig. 3, Panel A), the system still resists transitioning out of the isolated state. Although the trajectories show slight deviations due to noise, they quickly return to the isolated basin. Because $Z$ remains stable within the isolated state, the tipping point does not emerge.

#### Simulating a destabilizing state

In the destabilizing state simulation, the system becomes less resilient to the isolated state. The potential landscape (Fig. 2, Panel B) shows that the isolated attractor basin has become shallower, and a new attractor basin representing the healthy state has begun to emerge. A local maximum now appears between them, representing the tipping point and signifying an unstable equilibrium. Its presence indicates a shift toward *bistability*: the system now permits transitions between isolated and healthy states.

In the trajectory plot without perturbation (Left side of Fig. 3, Panel B), the system remains near the isolated state but shows drift toward the unstable region near the tipping point (displayed as a red dotted line). When a positive perturbation appears (Right side of Fig. 3, Panel B), three individual trajectories reach the tipping point and gravitate toward the healthy attractor. This result highlights how large perturbations, which were previously absorbed by the system, can now lead to qualitative shifts in state.

#### Simulating a tipping state

In the tipping point state (Fig. 2, Panel C), the isolated and healthy attractor basins are relatively shallow, and the ridge separating them—the tipping point—is less pronounced than in the destabilizing state. This flattened landscape suggests that the system has lost resilience and is now highly sensitive to small internal or external fluctuations. Minor changes in state are not strongly resisted, meaning that even subtle inputs can shift the system toward either attractor.

Trajectory simulations without perturbation (Left side of Fig. 3, Panel C) confirm that the system is near a tipping point. For the first time, some trajectories settle near the isolated attractor, while others drift toward the healthy attractor before perturbation, highlighting how small fluctuations represented by random noise can tip the system. When a positive perturbation is introduced (Right side of Fig. 3, Panel C), the precariousness of this tipping point state is further illustrated: several trajectories shift into the healthy attractor basin, while others remain near the unstable region.

#### Simulating a healthy state

The final simulation depicts a healthy state. As shown in the potential landscape (Fig. 2, Panel D), the system now exhibits a single deep attractor well on the right, corresponding to social connection. The system has high resilience, where the healthy state is the only stable equilibrium.

The trajectory plots further illustrate the reliance of the heathy state. Without perturbation (Left side of Fig. 3, Panel D), all trajectories remain near the healthy attractor throughout the entire simulation period. With a large negative perturbation (Right side of Fig. 3, Panel D), trajectories show only brief deviations before returning to the healthy state. Because $Z$ is stable within the healthy state, the tipping point does not emerge.

Although these simulations are valuable in illustrating the qualitative shifts in the equation dynamics, they do not test for parameter identifiability. That is, it is still unclear if the model can be uniquely learned from data. In the following section, we perform a simulation-recovery analysis to examine parameter identifiability.

### Parameter identifiability

We conducted a simulation–recovery analysis to evaluate whether the model parameters, including $a,{b},\,\sigma ,$ and the latent trajectory ${Z}_{t}$, are recoverable from different sampling designs. We (i) chose “ground-truth” parameters, (ii) generated data from the model, then (iii) fit the same model back to those data to see if we can recover the parameters and the latent trajectory. This analysis answers two identifiability questions that real data cannot: (1) *in principle*, if the model is correct, can the estimator find the right parameters? and (2) how do design choices (number of days, observations per day) affect accuracy? If recovery fails under known truth, failure with real data is uninterpretable; if recovery succeeds under realistic designs, inferences from real data are more defensible.

The first step was to formulate a measurement equation separate from our stochastic differential equation (see Eq. 4). A measurement equation is needed because we do not observe the latent process ${Z}_{t}$ directly. ${Z}_{t}$ is observed through collected data which contains noise, and we need a way to explicitly separate process noise from measurement noise. To do so, we used the following equation:

5 $$yt={Z}_{t}+{\varepsilon }_{t}$$

where ${\varepsilon }_{t}$ is a noise term with a normal distribution ${\varepsilon }_{t} \sim {{\mathscr{N}}}(0,{\tau }^{2})$. This specification explicitly separates process noise in the state equation ($\sigma$ $d{W}_{t}$, capturing unmodeled dynamics) from measurement noise ${\varepsilon }_{t}$ (with variance ${\tau }^{2}$). Estimating both $\sigma$ and $\tau$ prevents conflating within-system variability with measurement error.

#### Parameter estimation

We estimated parameters $\theta =(a,b,\sigma ,\tau )$ and the latent trajectory ${Z}_{t}$ within a nonlinear state–space using an Unscented Kalman Filter (UKF) for forward prediction–correction and a Rauch–Tung–Striebel (RTS) smoother for backward refinement. The UKF handles the cubic drift in Eq. (1) without linearization by propagating a small set of deterministically chosen sigma points through the nonlinear state updated over each observed time gap; the sigma points are then re-collapsed into a predicted mean and variance for ${Z}_{t}$. The RTS uses the full sequence of observations to refine earlier state estimates, yielding smoothed trajectories and uncertainty bands. Parameters are obtained by maximum likelihood using the UKF’s one-step-ahead innovations. At each time step, the filter predicts the next observation from the current state belief (based on Eqs. 1 and 2), compares this to the actual ${y}_{t}$, and accumulates a Gaussian log-likelihood from the innovation and its variance. We maximize this likelihood over $(a,b,\sigma ,\tau )$ using bounded multi-start optimization. For more information on the UKF, we refer readers to [49].

#### Simulation-recovery design

We conducted two recovery scenarios using simulated data which represented intensive longitudinal designs. The first scenario used 30 days × 3 observations/day (90 observations)—a sampling design often seen in the EMA literature. Given observations that dynamics in social isolation may emerge over longer time internals [25], the second scenario was a longer and denser design that used 365 days × 5 observations/day (1,825 observations). For both designs we drew 100 independent ground-truth parameter sets by sampling uniformly from ranges chosen to reflect plausible dynamics and noise: $a\in [1.0,\,1.6]$ (restoring strength spanning moderate to strong curvature), $b\in [-0.25,\,0.25]$ (tilt from slightly isolated to slightly healthy), $\sigma \in [0.25,\,0.45]$ (process noise consistent with day-to-day fluctuations), and $\tau \,[0.15,\,0.35]$ (observation noise typical of intensive longitudinal data). Each sampled parameter set defines one “truth,” from which we simulated a time series and then fit the model. Performance was summarized with root mean squared error (RMSE) and mean signed error (MSE) across the 100 replications. RMSE captures overall estimation error, in which lower RMSE values and values near 0 indicate better recovery. MSE quantifies systematic bias: values near zero indicate no consistent over- or under-estimation; negative values mean the estimator tends to undershoot, positive values mean it tends to overshoot.

#### Simulation-recovery results

The 365-day × 5/day design yielded markedly better recovery than the 30-day × 3/day design, especially for the drift parameters. For example, ${b}$ improved from RMSE = 0.46 (30 × 3) to 0.24 (365 × 5), and *a* improved from RMSE = 0.45 to 0.20. MSE also shrank: $a$ moved from a notable negative bias (−0.25) in the short design to essentially zero (+0.00) in the long design; $b$ was near-unbiased in both designs (−0.01 → +0.02). The noise terms were accurate in both scenarios, with *σ*RMSE 0.11 → 0.05 and *τ*RMSE 0.05 → 0.03. These results indicate that longer monitoring with more observations substantially improves identifiability of the landscape parameters (*a*, *b*), while the noise parameters ($\sigma ,\tau$) are recovered well even under shorter designs.

**Fig. 4:**
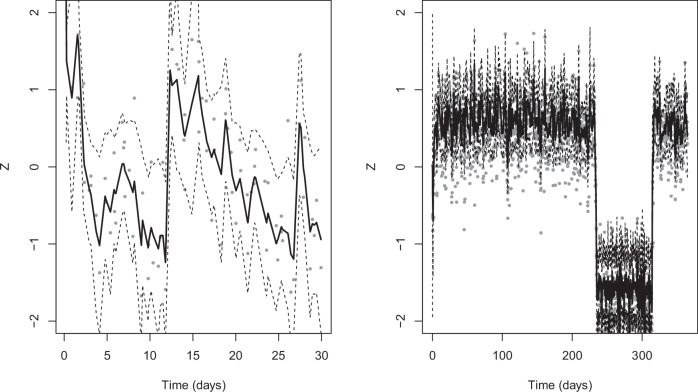
Recovery of the Latent State *Z*_*t*_. Observed values are plotted as gray points; the solid line shows the UKF/RTS–smoothed estimate of ${Z}_{t}$; and the bands depict pointwise 95% uncertainty intervals for the latent trajectory. The shorter design (left, 30 days × 3/day) tracks broad trends but with wider bands, whereas the longer, denser design (right, 365 days × 5/day) yields tighter intervals and closer tracking of the underlying state. For both designs, we chose the same parameter values from the ground-truth ranges ($a=1.2{;b}=-0.15;\sigma =0.35;\tau =0.25$).

Figure 4 illustrates recovery of the latent trajectory ${Z}_{t}$ from the two sampling designs using the UKF/RTS estimator. In the 30-day, 3/day design (left), the smoothed ${Z}_{t}$ tracks broad trends but shows noticeable lag and wider uncertainty bands, consistent with limited information per unit time. In the 365-day, 5/day design (right), the recovered trajectory hugs the observations more closely and the bands narrow, indicating greater certainty about state evolution. Put simply, longer monitoring with more prompts per day gives the filter more varied innovations to learn the cubic drift, resulting in more faithful reconstructions of ${Z}_{t}$. These visuals complement the parameter-recovery results: when $a$ (restoring strength) and $b$ (tilt) are better identified, the inferred state path is correspondingly more accurate.

This simulation–recovery analysis was intentionally narrow in scope. We examined a small set of sampling designs under one observation model and fixed parameter ranges. However, we did not explore subject heterogeneity, missingness patterns, alternative measurement noise structures, or model misspecification. A more comprehensive study is needed to refine thresholds for reliable recovery. Hence, we view these results as a proof-of-concept that the model dynamics are estimable and not necessarily as definitive design guidance.

### Identifying signs of state transition

Thus far, we have outlined how social isolation can be understood through a dynamical systems perspective, formalized this view with a general mathematical model, and provided evidence of parameter identifiability through a simulation-recovery analysis. Yet, none these procedures necessarily estimate resilience, which is a critical component in the proposed dynamics of social isolation. The resilience of attractor states can provide information on when individuals may be *vulnerable* to tipping points and help identify signs of transitions between states. A critical challenge in this framework is how to analyze (or approximate) resilience from intensive longitudinal data. One option to help identify resilience is *statistical process control* (SPC [50, 51]), a method from control theory that uses intensive longitudinal data to track system dynamics and potentially detect transitions between different system states.

**Fig. 5:**
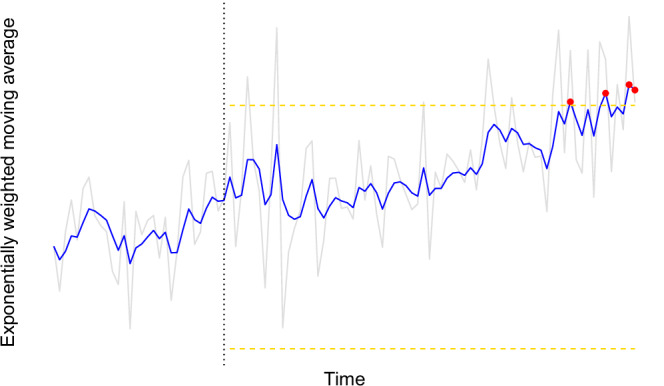
Illustrative Example of Statistical Process Control. Statistical process control uses the mean and standard deviation from the baseline phase (before the black dotted line) to calculate upper control limits (above the yellow dotted line). The blue line shows the exponentially weighted moving average of the variable over time, while the gray line represents the raw data. Values exceeding the control limit are flagged as red dots, indicating potential early signs of a transition to a different state.

SPC establishes *control limits*—statistically derived thresholds that define the expected range of variation in a variable during a baseline period. One approach is to use an exponentially weighted moving average (EWMA), which gives more weight to recent observations and allows the detection of gradual, sustained changes in the system [52]. Figure 5 shows a simulated, conceptual illustration. For example, if daily social interaction levels—or another variable that could approximate the temporal dynamics from the dynamical systems model—remain relatively steady over a few weeks, EWMA-based limits can be calculated to define an expected range. When future values fall outside these bounds, it may signal that the system is losing resilience and approaching a tipping point. Importantly, these control limits are person-specific, enabling the detection of individual deviations that would be missed by group-level models.

While SPC offers a promising approach to quantifying resilience in psychological systems, it has limitations—such as sensitivity to baseline length and defining appropriate control limits [53, 54]. In addition, SPC assumes a relatively stable baseline period, which may not always be the case. The best way to measure resilience in psychological systems remains an open question, and further methodological development is needed to capture the individualized nature of these dynamics.

## Discussion

Operationalizing social isolation as a complex dynamical system presents both substantive challenges and great possibilities for future research. One key challenge will be to identify which variables best function as internal and external parameters in these systems. Such variables may include behavioral and environmental features or neurophysiological variables that indicate sensitivity to social signals or stress [55]. Another challenge is clarifying the relevant timescales for these variables. Some psychological processes fluctuate rapidly within hours or days [49], while others exert influence over longer intervals [56]. Internal and external variables may also operate on different timescales. It is reasonable to assume that internal variables, such as social motivation, may fluctuate more rapidly than external variables, which may evolve more slowly over time (e.g., social network structure). Leveraging intensive longitudinal data and passive sensing, such as smartphone-based sensors [57], could clarify the time-varying nature of these variables and help identify risk and protective factors. For instance, burst designs with intensive repeated measurements can target risk factors that evolve rapidly, whereas passive sensing and longer EMA periods with sparse sampling (e.g., one assessment per day over a year) can target risk factors that evolve more slowly. Combining these different measurement designs would align data collection with our parameter-recovery findings. Model parameters were more reliably identified with more days and more observations per day, but collecting multiple observations daily for a full year or more is often infeasible. A hybrid design that embeds periodic bursts within longer, low-burden EMA and augments with passive sensing provides a practical compromise that approximates the needed time scale while keeping participant burden manageable.

The dynamical systems framework proposed here can be extended in numerous ways. One way it can be extended is to a multiple-subjects design. Although we took an inherently idiographic approach for our model simulations and parameter-recovery analysis, methodological advances in dynamical systems now allows for the modeling of random effects and person-level predictors of state transitions. This type of model was recently applied to substance use relapse, in which steepness and tilt (i.e., the $a$ and $b$ paramters) were allowed to vary across individuals and baseline variables were included as covariates [30]. Similar procedures could be applied to model social isolation, where, for instance, $a$ covaries with depression level and $b$ covaries with age or other relevant variables. Multiple-subjects designs can be implemented via the R package *dynr* [49], in which latent states are estimated based on Kalman filtering algorithms.

A second extension is to move beyond a single latent dimension. Our present 1-D model summarizes social isolation as one latent trajectory whose landscape is shaped by internal and external factors. Those factors could instead be operationalized as their own latent trajectories that co-evolve with social isolation in a 2-D or 3-D latent space, enabling explicit feedback among facets that likely operate on different timescales (akin to dynamics proposed in Toh et al.’s qualitative model of social isolation [25]). Accommodating such complexity achieves modeling that is better mapped to the multifaceted nature of isolation, yet the trade-off is that richer dynamics likely require more diverse measurement (e.g., multiple indicators per latent trajectory) to maintain identifiability and stable estimation. Accordingly, we view the 1-D model as a parsimonious baseline and higher-dimensional systems as a clear next step for future work.

Viewing social isolation as a dynamical system also opens new opportunities for early identification and targeted intervention, especially for those at risk for serious mental illness. Social withdrawal and reduced social motivation often precede the onset of psychosis or depression [58, 59], suggesting that early changes in behavior may reflect a system nearing a tipping point. For example, a decline in interaction frequency or an increase in avoidance could signal a shift toward an isolated state. By using intensive longitudinal data from ecological momentary assessment or passive sensing, SPC could detect early signs of reduced resilience before clinical symptoms fully emerge. Rather than waiting for symptoms to meet diagnostic thresholds, predictive monitoring allows for earlier intervention before major problems emerge, especially if deployed at clinically strategic times (e.g., discharge from intensive treatment program, during stressful life transition). If SPC detects a drift toward a socially isolated state, clinicians could, for example, introduce cognitive-behavioral therapy or motivational interviewing remotely to boost engagement and reduce avoidance. These interventions could be adjusted dynamically based on ongoing data, making it possible to modulate treatment intensity in real time [52, 60]. Such proactive, tailored care could help prevent individuals from tipping into an isolated state and reduce the risk of progression to psychiatric disorders.

Despite growing recognition of the health consequences of social isolation, and its links to early mortality, our understanding of its nature and why it may become chronic remains limited. Most existing models treat isolation as a static condition. In contrast, the dynamical systems framework presented here conceptualizes social isolation as an emergent, self-reinforcing process shaped by evolving internal and external forces. This framework not only enables more precise, testable hypotheses about the development of social isolation but also has the potential to inform real-time interventions to mitigate the onset of both isolation and associated psychiatric illness.

## Data Availability

No human subject or animal data were used in this manuscript. The R code to reproduce all the simulations and results of this paper can be found at https://osf.io/a2qg9/.

## Footnotes

1 Resilience is distinct from *stability*, which is defined as the speed at which a system recovers to its equilibrium after small perturbations [33]. While both are relevant in the dynamical systems perspective proposed here, we focus on resilience rather than stability because resilience captures not only the system’s tendency to return to a given state but also its capacity to withstand perturbations without shifting into a different state.

2 In dynamical systems theory, external and internal control variables are also referred to as “splitting” and “normal” variables, respectively [40].

3 The fractions in the equation are the antiderivatives chosen to cancel the factors that appear on differentiation. Using ¼ and ½ avoids extra 4’s and 2’s in the drift; it’s a standard normalization so $\frac{{dV}}{{dZ}}={Z}^{3}-{aZ}-b$ and thus $-\frac{{dV}}{{dZ}}=-{Z}^{3}+{aZ}+b$.

4 For illustrative purposes, values of the initial state variable $Z,$ internal control parameter $a$, external control parameter $b,$ and stochastic noise variable $\sigma$ were varied across simulations to demonstrate the different system regimes.
